# Diagnostic accuracy of perioperative electromyography in the positioning of pedicle screws in adolescent idiopathic scoliosis treatment: a cross-sectional diagnostic study

**DOI:** 10.1186/s12891-020-03491-z

**Published:** 2020-07-20

**Authors:** Carlos Eduardo Barsotti, Bruno Moreira Gavassi, Francisco Eugenio Prado, Bernardo Nogueira Batista, Raphael de Resende Pratali, Ana Paula Ribeiro, Carlos Eduardo Soares de Oliveira, Ricardo Rodrigues Ferreira

**Affiliations:** 1 Spine Group, Institute of Medical Assistance to the State Public Hospital Servant – IAMSPE, Centro de Estudos de Ortopedia, Rua Borges Lagoa, 1755, 1 andar – sala 180, São Paulo, SP CEP: 04038-034 Brazil; 2 School of Medicine, University of Sao Paulo, São Paulo, SP Brazil

**Keywords:** Scoliosis, Bone screw, Pedicle screw, Electromyography, Intraoperative neurophysiological monitoring, Computed tomographic scan

## Abstract

**Background:**

To investigate in the conventional techniques of the pedicle screws using triggered screw electromyography (t-EMG), considering different threshold cutoffs: 10, 15, 20 25 mA, for predicting pedicle screw positioning during surgery of the adolescent with idiopathic scoliosis (AIS).

**Methods:**

Sixteen patients (4 males, 12 females, average age 16.6 years) were included, with an average curve magnitude of 50 degrees and placement of 226 pedicle screws. Each screw was classified as “at risk for nerve injury” (ARNI) or “no risk for nerve injury” (NRNI) using CT and the diagnostic accuracy of EMG considering different threshold cutoffs (10,15, 20 and 25 mA) in the axial and Sagittal planes for predicting screw positions ARNI was investigated.

**Results:**

The EMG exam accuracy, in the axial plane, 90.3% screws were considered NRNI. In the sagittal plane, 81% pedicle screws were considered NRNI. A 1-mA decrease in the EMG threshold was associated with a 12% increase in the odds of the screw position ARNI. In the axial and sagittal planes, the ORs were 1.09 and 1.12, respectively. At every threshold cutoff evaluated, the PPV of EMG for predicting screws ARNI was very low in the different threshold cutoff (10 and 15); the highest PPV was 18% with a threshold cutoff of 25 mA. The PPV was always slightly higher for predicting screws ARNI in the sagittal plane than in the axial plane. In contrast, there was a moderate to high NPV (78–93%) for every cutoff analyzed.

**Conclusions:**

EMG had a moderate to high accuracy for positive predicting value screws ARNI with increase threshold cutoffs of 20 and 25 mA. In addition, showed to be effective for minimizing false-negative screws ARNI in the different threshold cutoffs of the EMG in adolescent with idiopathic scoliosis (AIS).

## Background

Pedicle screw fixation plays an important role in spine surgery due to its firm three column control and thus provide superior reconstruction stability. As a common surgical procedure, the pedicle screw placement method has been widely employed to reconstruct local stability in spine surgery due to its great three-column control [1]. The use of pedicle fixation is increasing in spinal arthrodesis and is especially common in the treatment of adolescent idiopathic scoliosis (AIS). First demonstrated in the lumbar segments of the spine [2, 3], the technique has also been proven to be safe when employed at thoracic levels [4–7].

Variety of techniques have been introduced to assist screw insertion and to reduce the prevalence of pedicle violation, given the clinical complications that may occur during and after the surgical procedure. A misplaced pedicle screw may result in neurovascular damage, dural tearing, or visceral involvement, and such complications are potential threats to life and limb [8]. The conventional free-hand technique is currently employed with the fluoroscopy-guided method as the primary method of pedicle screw implantation. However, the accuracy of pedicle screw placement using conventional techniques is limited by the operator’s field of vision and uncertain factors, such as individual differences and changes in body position [1, 8].

Recently, robot-assisted systems have been developed to address the issue of pedicle screw malposition. Many retrospective studies consistently reported that the clinically acceptable screw positioning accuracy under robotic guidance is near 99% [9, 10]. Two prospective, randomized, controlled trials demonstrated that robot-assisted pedicle fixation has the same pedicle screw placement accuracy as and even less than that of the freehand conventional technique [11, 12]. However, some researchers revealed through a meta-analysis that the robot-assisted pedicle screw insertion technique has no significant advantage over the conventional technique [13, 14], but another meta-analysis demonstrated that the robot-assisted technique is superior to the conventional method in terms of pedicle screw placement accuracy [15], because this associated to fewer proximal facet joint violation and less intraoperative radiation exposure, however, longer surgical duration than freehand technique [1]. Controversy remains as to whether robot-assisted techniques are more accurate in pedicle screw placement compared with the conventional freehand technique.

Despite this controversy, several literature studies reveal that the instrumentation with pedicle screws allows for better correction of spinal deformities in the coronal, sagittal and rotational planes, less correction loss, shorter constructions and improved lung function without increasing neurological complications [16, 17]. Pedicle screw misplaced rates using conventional techniques ranged from 5 to 41% in the lumbar spine and from 3 to 55% in the thoracic spine [18, 19], with an estimated 1% rate of neurologic complication. Triggered screw electromyography (t-EMG) has been used to help identify misplaced screws, with threshold stimulation varying based upon the spinal segment tested (lumbar versus thoracic), location within the curve (apical versus non-apical) and laterality with regard to the curve (concavity versus convexity) [20, 21].

Due to peculiar vascular and neurologic anatomical features of the vertebrae and spinal canal, caution is needed during insertion of pedicle screws. For safety reasons, it is of utmost importance to ensure precise insertion and to confirm an intraosseous position [22]. Intraoperative neurophysiologic monitoring during pedicle instrumentation allows the early detection and prevention of neurological complications [23, 24]. Motor evoked potentials, somatosensory evoked potentials, free-running electromyography (EMG) and stimulated EMG can be used as monitoring techniques. Furthermore, stimulated EMG can be applied directly to the inserted screws [20].

In patients with scoliosis, rotational deformity increases the risk of perforating the pedicle cortical wall, which is not always easy to detect during surgical procedures. Intraoperative assessment of the position of the screws with conventional radiography or fluoroscopy can help detect misplaced implants, although computed tomography (CT) provides greater accuracy [25]. EMG can provide additional information to establish a link between the implants and neural elements. However, the diagnostic accuracy of intraoperative EMG to detect pedicle screw malposition is not well understood. Thus, the aim of this study was to investigate in the conventional techniques of the pedicle screws using triggered screw electromyography (t-EMG), considering different threshold cutoffs: 10, 15, 20 25 mA, for predicting pedicle screw positioning during surgery of the adolescent with idiopathic scoliosis (AIS). The initial hypothesis was that lower EMG thresholds would be observed for screws at higher risk for nerve injury.

## Methods

### Design, setting, participants and ethics

This was a prospective study (cross-sectional type) involving patients with AIS who underwent surgical treatment in the same hospital and in whom intraoperative EMG measurements were compared with the implant positions evaluated by CT. The study was reviewed and approved by the Departmental Research Committee of the Institute of Medical Assistance to the State Public Hospital Servant – IMASPS (registration number: 533.892), in accordance with relevant guidelines and regulations. All participants provided their informed consent by written underwent assessment and experimental procedure.

Sixteen patients (4 males, 12 females, average age 16.6 years) who underwent surgery performed by the same surgical team in the same institution from March to December 2013 were included in the study, with an average curve magnitude of 62 degrees and placement of 226 pedicle screws to attain an average curve correction of 77.7%. Patients were excluded if they had scoliosis with a known etiology (i.e., not AIS), if they were undergoing revision surgery, or if no postoperative CT was available for review.

All patients were operated by a posterior approach, with insertion of pedicle screws from the same manufacturer (DePuy, Synthes, Raynham, MA, USA) by the “free hand” technique [22] and under intravenous anesthesia [26]. Neurophysiological monitoring was employed in all surgeries using the same technique and device.

### Variables and measurements

After pedicle screw insertion, the stimulation was performed using EMG with monopolar electrode (cathode) and a subdermal needle electrode inserted into the paravertebral musculature (anode). Stimulation was performed with a frequency of 3 Hz, a duration of 0.1 ms and an increasing intensity until an EMG response could be observed. The maximum intensity used was 30 m-amperes (mA). For each screw, the lowest intensity able to generate a measurable response was recorded as the EMG threshold for that screw. If no response was observed, a value of 30 was assigned to that screw.

Pedicle screw stimulation was performed using DS7A current stimulation (Digitimer North America, LLC; Fort Lauderdale, FL) to generate and deliver square wave constant-current pulses to each pedicle screw following insertion. We used repetitive 4-pulse trains, applied with an interpulse interval of 2 msec between trains and an intertrain rate of 3 Hz. Maximal stimulus delivered varied between cases with maximums of 20, 30, or 40 mA. Minimum stimulus intensities were recorded for each level with a pedicle screw. All screws demonstrating a minimum intensity ≥30 mA was defined as a maximal intensity. Only one patient was classified as having a threshold ≤8 mA was defined as abnormal and underwent screw re-direction, but underwent screw removal and probing of the pedicle track using a ball-tip probe. This information has been clarified in the text for better understanding [20].

In the EMG exam was considering different threshold cutoffs: 10, 15, 20 and 25 in the axial and sagittal planes to analysis the accuracy after AIS surgery. All patients performed the computed tomography (CT) exam, immediately after the surgery before the patient performs the gait, to evaluate the implant positioning and classification according to the criteria proposed by Abul-Kasim et al. [27]. This grading system was developed to distinguish between lateral, medial and anterior cortical perforations and foraminal perforation and is based on whether the cortical violation is partial or total rather than the length (mm) of the perforation.

**Fig. 1:**
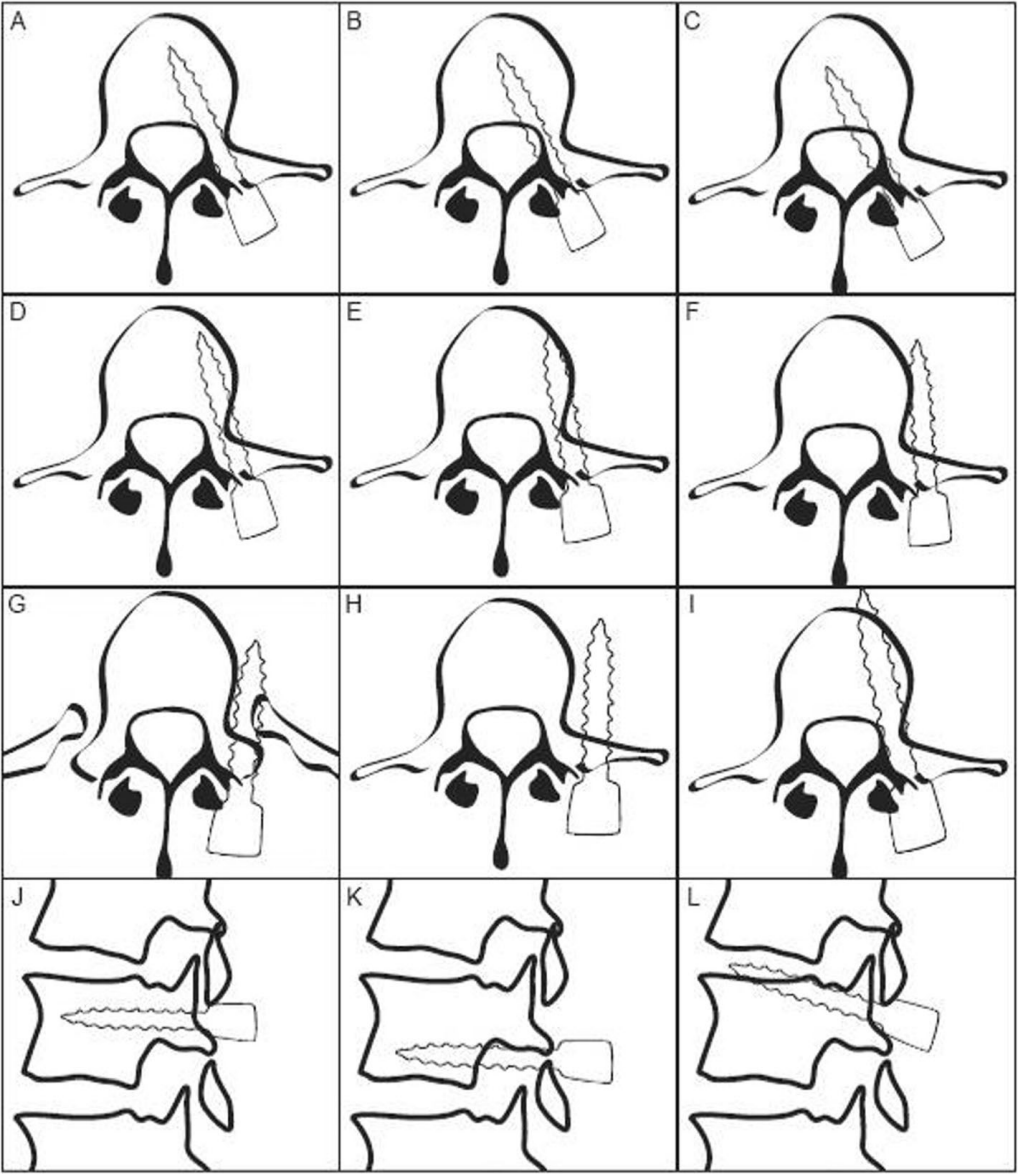
Different types of misplacement according to the here proposed grading system. **a**–**f** axial images and (**g**–**i**) sagittal images. **a**: Acceptably placed pedicle screw. **b**: MCP grade 1. **c**: MCP grade 2. **d**: LCP grade 1. **e**: LCP grade 2. **f**: ACP. **g**: Acceptably placed pedicle screw on a sagittal image with no FR or EPP. **h**: FP. Perforation into the underlying neural foramen. **i**: EPP. Perforation through the upper endplate. Drawing done by Abul-Kasim, K. (2009). *Adolescent Idiopathic Scoliosis. The Role of Low Dose Computed Tomography.* Department of Radiology, Lund University

**Fig. 2:**
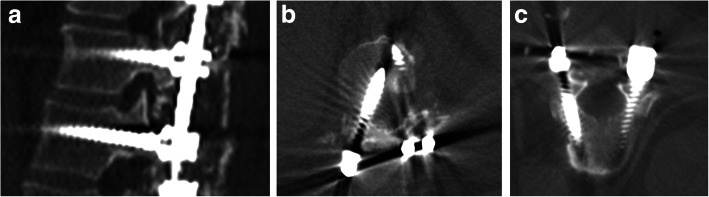
The pedicle was assessed and classified in both the medial and sagittal planes as follows: normally placed in the medial plane; medial cortical perforation (MCP) grade 1, partially medialized; MCP grade 2, totally perforating the medial pedicular cortex; lateral cortical perforation (LCP) grade 1, partially lateralized but anchored in the vertebral body; LCP grade 2, abutting the outer cortex of the vertebral body and not anchored in the vertebral body; normally placed in the sagittal plane; perforating the inferior underlying neural foramen (INF); or perforating the superior underlying neural foramen (SUP)

Each individual screw to the corresponding pedicle was assessed and classified in both the medial and sagittal planes as follows: normally placed in the medial plane; medial cortical perforation (MCP) grade 1, partially medialized; MCP grade 2, totally perforating the medial pedicular cortex; lateral cortical perforation (LCP) grade 1, partially lateralized but anchored in the vertebral body; LCP grade 2, abutting the outer cortex of the vertebral body and not anchored in the vertebral body; normally placed in the sagittal plane; perforating the inferior underlying neural foramen (INF); or perforating the superior underlying neural foramen (SUP). Additionally, screws classified as MCP grade 1 or MCP grade 2 in the axial plane and as perforating the INF or the SUP in the sagittal plane were considered “at risk for nerve injury” (ARNI), as these screws are closer to neural elements. Screws classified as normally placed and screws classified as LCP, although recognized as misplaced, were considered “no risk for nerve injury” (NRNI) (Figs. 1 and 2).

### Bias control

The total intravenous technique (TIVA) was used to induce anesthesia in the AIS surgeries, and medications that usually do not interfere with intraoperative neurophysiological monitoring (propofol and remifentanil) were administered. All AIS corrections included in this study were performed by the same surgical team, using the same techniques for surgery and electromyography evaluation. All CT scans were evaluated by the same observer (BMG).

### Statistical analysis

The sample size calculation on 16 patients (75% female and 25% male) was based on the mean age and Cobb angle preoperative, using the G-Power 3.0 software, and considered a moderate effect size (*F* = 0.25), a power of 80%, and a significance level of 5%”. Continuous variables are presented as the mean and standard deviation, and categorical data are presented as absolute and relative frequencies. A descriptive analysis of the positioning of all screws was performed. To evaluate the diagnostic accuracy of EMG for predicting screws ARNI, we excluded all screws inserted above T6, as those pedicles have lower reliability for EMG acquisition [28]. A single patient contributed multiple sampling units (screws) to the analysis, resulting in a hierarchical structure of the generated data, with subjects as the primary sampling units and individual screws as the secondary sampling units. The association between the EMG threshold recorded intraoperatively and postoperative screw status, considering the risk for nerve injury, was investigated using generalized estimating equations (GEEs). Only medial cortical perforation of the screw (MCP grade 1 or MCP grade 2) was considered a positive outcome (ARNI) in the axial plane, while both superior or inferior deviation of the screw were considered positive outcomes in the sagittal plane. These statistical models are similar to logistic regression models but take into account the hierarchical structure of the data.

The diagnostic accuracy of EMG for predicting screw malposition was investigated using a receiver operating characteristic (ROC) curve. The curve represents a plot of the sensitivity and specificity at progressive cutoffs of a diagnostic test measured on a continuous scale. Therefore, the area under the curve (AUC) is a measure of the ability of EMG to discriminate between screws ARNI and screws NRNI. Estimates of diagnostic accuracy, including sensitivity (Sn), specificity (Sp), negative predictive value (NPV) and positive predictive value (PPV), were calculated for cutoffs at every 5 mA and are presented with their 95% confidence intervals (95%CI). All statistical analyses were performed using the statistical package STATA 14 (StataCorp, TX/EUA). Associations with *p* < .05 (two-sided) were considered significant.

## Results

In the study period, 16 patients underwent surgical treatment for AIS and were included in this study. Most (*n* = 12, 75% female and *n* = 4, 25% male) were female, and the average age was 16.6 years. No patient had neurological complaints, experienced irradiated pain to dermatomes or exhibited any observed change in the physical exam indicating nerve injury.

A total of 281 pedicles were analyzed for screw position. In the axial plane, 195 screws were in the ideal position (69.4%). There was lateral cortical perforation in cases of which 25 (8.9%) were classified as LCP1, and 16 (5.7%), as LCP2. Medial cortical perforation was found in 45 cases (27 [9.6%] were MCP1, and 18 [6.4%] were MCP2) (Table 1, supplementary data.).

In the sagittal plane, 226 pedicle screws were in the ideal position (80.4%), while 48 (17.1%) violated the inferior foramen (FP1 INF), and 7 (2.5%), the superior foramen (FP1 SUP). Considering axial and sagittal planes together, 59.1% (166/281) of all screws had no cortical perforation (lateral, medial or superior or inferior foramens), representing ideal positions, while 40.9% (115/281) of screws showed at least one degree of cortical perforation (Table 2, supplementary data).

Below T6, 226 pedicles were considered in the EMG accuracy study (Table 1). In the axial plane, 204 (90.3%) screws were considered NRNI, of which 136 (60.2%) were ideally positioned and 68 (30.1%) had LCP, and 22 (9.7%) screws were considered ARNI with MCP. In the sagittal plane, 183 (81%) pedicle screws were considered NRNI, while 43 (19%) violated the inferior foramen (FP1 INF) or the superior foramen (FP1 SUP) and were considered ARNI.

**Table 1:** Pedicle screws considered in the EMG accuracy diagnostic study

***Total***	***N=***	***100%***	
***Axial Plane***	**N**	**%**	
LCP0	36	15.9	NRNI - 204 (90.3%)
LCP1	19	8.4	
LCP2	13	5.8	
MCP0	136	60.2	
MCP1	16	7.1	ARNI - 22 (9.7%)
MCP2	6	2.6	
***Sagittal Plane***	**N**	**%**	
FP0	183	81	NRNI - 183 (81%)
FP1 (SUP)	38	16.8	ARNI - 43 (19%)
FP1 (INF)	5	2.2	

Legend: *MCP* medial cortical perforation, *LCP* Lateral cortical perforation, *FP0* posterior foramen, *FP1 INF* inferior foramen (FP1 INF) and *FP1 SUP* superior foramen (FP1 SUP), *ARNI* At risk for nerve injury, *NRNI* No risk for nerve injury, *INF* Inferior underlying neural foramen, *SUP* Superior underlying neural foramen

We observed a statistically significant association between EMG responses at lower intensities and the positioning of screws associated with risk for nerve injury. Overall, a 1-mA decrease in the EMG threshold was associated with a 12% increase in the odds of the screw position ARNI (OR = 1.12; 95% CI = 1.06–1.18; *p* < .001). In the axial and sagittal planes, the ORs were 1.09 (95% CI = 1.03–1.16; *p* = .005) and 1.12 (95%CI = 1.04–1.2; *p* = .004), respectively. However, the ROC curves showed that EMG had moderate ability to discriminate between screws ARNI and NRNI.

The AUCs for the axial, sagittal and combined planes were .65 (95% CI = .57–.74), .63 (95% CI = .51–.75) and .65 (95% CI = .55–.75), respectively. Table 2 shows the performance estimates of EMG for predicting screws positioned ARNI in our sample. At every threshold cutoff evaluated, the PPV of EMG for predicting screws ARNI was very low; the highest PPV was 18% with a threshold cutoff of 25 mA. The PPV was always slightly higher for predicting screws ARNI in the sagittal plane than in the axial plane. In contrast, there was a moderate to high NPV (78–93%) for every cutoff analyzed (Table 2).

**Table 2:** EMG accuracy as a diagnostic criterion considering different threshold cutoffs

EMG Threshold Cutoff (mA)	OVERALL	AXIAL	SAGITAL
10	Sn: 18% (9–30%)	Sn: 14% (3–35%)	Sn: 21% (10–36%)
	Sp: 100% (98–100%)	Sp: 97% (93–99%)	Sp: 99% (97–100%)
	NPV: 78% (72–83%)	NPV: 90% (86–94%)	NPV: 83% (78–88%)
	**PPV: 10%k (5–17%)**	**PPV: 3% (1–8%)**	**PPV: 9% (4–16%)**
15	Sn: 38% (25–51%)	Sn: 32% (14–55%)	Sn: 42% (27–58%)
	Sp: 85% (78–90%)	Sp: 80% (74–86%)	Sp: 84% (78–89%)
	NPV: 80% (73–86%)	NPV: 91% (86–95%)	NPV: 86% (80–90%)
	**PPV: 15% (10–22%)**	**PPV: 5% (2–10%)**	**PPV: 13% (8–20%)**
20	Sn: 48% (35–62%)	Sn: 50% (28–72%)	Sn: 49% (33–65%)
	Sp: 72% (65–79%)	Sp: 69% (62–75%)	Sp: 71% (64–77%)
	NPV: 81% (74–87%)	NPV: 93% (87–96%)	NPV: 86% (79–91%)
	**PPV: 16% (11–23%)**	**PPV: 7% (3–11%)**	**PPV: 13% (8–18%)**
25	Sn: 70% (56–81%)	Sn: 68% (45–86%)	Sn: 67% (51–81%)
	Sp: 48% (40–55%)	Sp: 45% (38–52%)	Sp: 46% (39–53%)
	NPV: 83% (74–90%)	NPV: 93% (86–97%)	NPV: 86% (77–92%)
	**PPV: 18% (13–23%)**	**PPV: 7% (4–11%)**	**PPV: 13% (9–18%)**

Legend: *Sn* sensitivity, *Sp* specificity, *NPV* negative predictive value and *PPV* positive predictive value

## Discussion

AIS is a complex three-dimensional deformity associated with rotation and structural abnormalities of the vertebrae, making treatment technically challenging. Implants for pedicle fixation have been widely used in surgical treatments of the thoracic and lumbar spine, with better results in arthrodesis rate, correction power and early mobilization of the patient compared with fixation systems that employ hooks or mixed systems [29, 30]. Pedicle screw misplacement is detected in 3 to 44% of cases in the literature [31, 32], and in this study, the rate was 40.9%. The differential of this study was a to analyze the occurrence of pedicle screw misplacement in a specific pathology, AIS using a conventional method with less cost and great access of patients with low socioeconomic status. Additionally, we evaluated the EMG as a method to electrically stimulate the positioned pedicle screw to assess its proximity to nearby nerve roots, considering different threshold cutoffs, as a diagnostic tool to predict screws ARNI in AIS surgery.

Screws with LCP are associated with risk of vascular or visceral damage [3, 33, 34]. In the present series, 14.6% of screws had LCP, in line with the literature [3, 33, 34], and no cases were associated with complications. For MCP, misplacement rates of 1.4 to 14% have been reported in the literature [3, 34], reaching 28% in one series [35]. In the present study, 16% of screws had MCP according to postoperative CT. There are limited data in the literature describing the misplacement of pedicle screws in the sagittal plane with superior or inferior cortical perforation. The literature many studies also clearly indicate a support for the robotic-assisted technique in the accuracy of pedicle screw placement [15], however, some authors hold the opposite opinion [11, 12]. The randomized controlled trial by Ringel et al. (2012) [12] demonstrated significantly poor screw insertion in the robotic-assisted technique compared with the free-hand with fluoroscopy-guided (85% vs 93%). Nevertheless, a meta-analysis by Liu et al. (2016) [16] pooled 3 RCTs and 2 cohort studies to address this controversy and demonstrated that no significant difference was found between the 2 techniques in terms of accuracy; therefore, it would require further studies to determine the unresolved clinical equipoise in this field. In addition, recent meta-analysis studies show that the robot-assisted technique is more accurate in pedicle screw placement than the freehand technique conventional method. The robot-assisted technique was associated with equivalent accuracy of pedicle screw implantation, less proximal facet joint violation, less intraoperative radiation exposure but longer surgical duration than freehand technique [1, 15, 19, 20]. The differential of the present study was to verify the use of electromyography (EMG), considering threshold cutoffs between 20 and 25 mA as effective for predicting pedicle screw positioning and lower risk for nerve injury during conventional surgery, by freehand technique, in adolescent with idiopathic scoliosis (AIS).

Intraoperative neurophysiology evaluation can allow the early detection and correction of possible lesions during spinal surgery. Such techniques include the evaluation of motor evoked potentials, somatosensory evoked potentials and EMG [23, 24]. The role of the EMG stimulus in the early identification of pedicle cortical perforation has been established for lumbar pedicles. Thresholds below 4 or 5 mA are suggestive of perforation [23], while thresholds above 15 mA indicate correct positioning of screws [34]. However, the correlation between EMG thresholds and screw positioning in thoracic pedicles has not yet been well established.

In evaluating the accuracy of EMG as an intraoperative diagnostic method to detect misplaced screws ARNI, there was a statistically significant association between EMG responses and the positioning of screws associated with risk for nerve injury. A decreased EMG threshold was associated with an increased odds of the screw position ARNI among thoracic and lumbar screws. The association between EMG threshold and screw misplacement in thoracic pedicles was recently shown using pulse-train stimulation [35]. The previous study only evaluated the association between EMG stimulation and screw position in the axial plane with MCP, while the present study evaluated both the axial and sagittal planes. Somatosensory and motor evoked potentials have been found to be both sensitive (95%) and specific (99.8%) for identifying significant sensory and motor nerve deficits during surgery [35], however, these do not necessarily identify malpositioned pedicle screws unless they impart direct spinal cord trauma. Given the limitation sensory and motor nerve deficits for identifying malpositioned pedicle screws, t-EMG was developed as a method to electrically stimulate the positioned pedicle screw to assess its proximity to nearby nerve roots [36]. Study have investigated the reliability of t-EMG for locating malpositioned screws, identifying a specificity of 0.94 [37].

Despite the association found between EMG and the position of screws, the ability of EMG to intraoperatively discriminate between screws ARNI and NRNI was poor to moderate. NPV and PPV are the most meaningful measures of diagnostic accuracy in terms of making clinical decisions based on a test result. NPV expresses the probability of not having the condition under study given a negative test outcome, and PPV expresses the probability of having the condition given a positive test outcome. EMG showed a very low PPV (< 18%) at every threshold cutoff evaluated, meaning that less than 1 out of 5 screws that test positive (achieving an electrical response at a threshold lower than the cutoff) would actually be positioned ARNI.

Considering the risk for neurological deficit or stenosis of the spinal canal if a screw breaches the medial wall during thoracic pedicle screw instrumentation in AIS surgery, the consequences of a false-negative result of a diagnostic test for screw malposition can be severe [38]. Therefore, it is imperative that the diagnostic test detect true positives and minimize false negatives, as represented by a high NPV. The present study revealed a moderate to high NPV of EMG as diagnostic test for every cutoff analyzed, and thus, EMG may be considered an accurate test to minimize false-negative screws ARNI.

The main limitation of the present study is that despite the aim to analyze the ability of EMG to intraoperatively predict screws ARNI, the sample was composed exclusively of patients with no nerve injury, spinal cord injury, or nerve root injury. Some false-positive and false-negative cases were found among the EMG responses of the diagnostic test, but these cases did not result in any clinical consequence. Therefore, it is not possible to assume that EMG is not an accurate tool for predicting screws ARNI. Furthermore, we consider intraoperative neurophysiologic monitoring, particularly motor evoked potentials, as extremely important during thoracic screw insertion for the early detection and prevention of severe neurological complications.

## Conclusions

EMG had a moderate to high accuracy for positive predicting value screws ARNI with increase threshold cutoffs 20 and 25 mA. In addition, showed to be effective for minimizing false-negative screws ARNI.

## Supplementary information

**Additional file 1: Table 1.** Summary of the positions of the pedicle screws in the axial and sagittal planesmfor each patient and the total pedicles analyzed. **Table 2.** Summary of the ideal screw positioning in the sagittal and axial planes and inadequate positioning in any of the planes.

## Data Availability

The datasets used and/or analysed during the current study are available from the corresponding author (cadubarsotti@gmail.com) on reasonable request.

## Abbreviations

EMG: Electromyography
AIS: Adolescent idiopathic scoliosis
CT: Computer Tomography
ARNI: At risk for nerve injury
NRNI: No risk for nerve injury
ROC: Receiver operating characteristic
MCP: Medial cortical perforation
LCP: Lateral cortical perforation
INF: Inferior underlying neural foramen
SUP: Superior underlying neural foramen
TIVA: Total intravenous technique (TIVA)
