# Supplementary material for: Diagnostic accuracy of perioperative electromyography in the positioning of pedicle screws in adolescent idiopathic scoliosis treatment: a cross-sectional diagnostic study
Source: BMC Musculoskelet Disord. 2020 Jul 20;21:473. doi: 10.1186/s12891-020-03491-z (PMC7372782; doi:10.1186/s12891-020-03491-z)
Supplement: Supplementary file 1 — Additional file 1: Table 1. Summary of the positions of the pedicle screws in the axial and sagittal planesmfor each patient and the total pedicles analyzed. Table 2. Summary of the ideal screw positioning in the sagittal and axial planes and inadequate positioning in any of the planes. [file 12891_2020_3491_MOESM1_ESM.pdf]

TABLES -

Table 1 Summary of the positions of the pedicle screws in the axial and sagittal planesmfor each patient and the total pedicles analyzed.

Axial plane

Patient	LCP 2		LCP 1		LCP 0/MCP 0		MCP 1		MCP 2		Total
	n	%	n	%	n	%	n	%	n	%	
1	4	15.4	0,	0.0	17	65.4	3	11.5	2	7.7	26
2	0	0.0	2	10.5	14	73.7	3	15.8	0	0.0	19
3	1	6.7	1	6.7	13	86.7	0	0.0	0	0.0	15
4	0	0.0	1	6.2	9	56.2	3	18.8	3	18.8	16
5	0	0.0	2	9.1	15	68.2	3	13.6	2	9.1	22
6	0	0.0	0	0.0	10	55.6	6	33.3	2	11.1	18
7	1	6.2	2	12.5	10	62.5	1	6.2	2	12.5	16
8	1	5.0	3	15.0	13	65.0	1	5.0	2	10.0	20
9	1	8.3	2	16.7	8	66.7	1	8.3	0	0.0	12
10	2	11.1	1	5.6	12	66.7	2	11.1	1	5.6	18
11	1	5.6	3	16.7	12	66.7	1	5.6	1	5.6	18
12	2	11.1	2	11.1	11	61.1	2	11.1	1	5.6	18
13	0	0.0	2	10.5	14	73.7	1	5.3	2	10.5	19
14	3	16.7	3	16.7	12	66.7	0	0.0	0	0.0	18
15	0	0.0	1	7.1	13	92.9	0	0.0	0	0.0	14
16	0	0.0	0	0.0	12	100.0	0	0.0	0	0.0	12
**Total**	**16**	**5.7**	**25**	**8.9**	**195**	**69.4**	**27**	**9.6**	**18**	**6.4**	**281**

Sagittal plane

Patient	FP 1 (inf.)		FP 0		FP 1 (sup.)		Total
	n	%	n	%	n	%	
1	0	0,0	26	100.0	0	0.0	26
2	1	5.3	17	89.5	1	5.3	19
3	4	26.7	11	73.3	0	0.0	15
4	6	37.5	10	62.5	0	0.0	16
5	1	4.5	18	81.8	3	13.6	22
6	9	50.0	9	50.0	0	0.0	18
7	4	25.0	12	75.0	0	0.0	16
8	0	0.0	18	90.0	2	10.0	20
9	0	0.0	12	100.0	0	0.0	12
10	5	27.8	13	72.2	0	0.0	18
11	8	44.4	10	55.6	0	0.0	18
12	6	33.3	12	66.7	0	0.0	18
13	2	10.5	17	89.5	0	0.0	19
14	0	0.0	18	100.0	0	0.0	18
15	1	7.1	13	92.9	0	0.0	14
16	1	8.3	10	83.3	1	8.3	12
**Total**	**48**	**17.1**	**226**	**80.4**	**7**	**2.5**	**281**

Legend: MCP - medial cortical perforation, LCP - Lateral cortical perforation, FP - posterior foramen, FP1 INF - inferior foramen (FP1 INF) and FP1 SUP - superior foramen (FP1 SUP).

Table 2 Summary of the ideal screw positioning in the sagittal and axial planes and inadequate positioning in any of the planes.

Patient	FP 0 and LCP 0/MCP 0		FP or LCP/MCP≠0		Total
	n	%	n	%	
1	17	65.4	9	34.6	26
2	13	68.4	6	31.6	19
3	10	66.7	5	33.3	15
4	7	43.8	9	56.2	16
5	14	63.6	8	36.4	22
6	6	33.3	12	66.7	18
7	6	37.5	10	62.5	16
8	13	65.0	7	35.0	20
9	8	66.7	4	33.3	12
10	9	50.0	9	50.0	18
11	8	44.4	10	55.6	18
12	8	44.4	10	55.6	18
13	13	68.4	6	31.6	19
14	12	66.7	6	33.3	18
15	12	85.7	2	14.3	14
16	10	83.3	2	16.7	12
**Total**	**166**	**59.1**	**115**	**40.9**	**281**

Legend: MCP - medial cortical perforation, LCP - Lateral cortical perforation, FP0 - posterior foramen.
